# Procalcitonin and mortality in status epilepticus: an observational cohort study

**DOI:** 10.1186/s13054-015-1072-9

**Published:** 2015-10-09

**Authors:** Raoul Sutter, Martina Valença, Sarah Tschudin-Sutter, Stephan Rüegg, Stephan Marsch

**Affiliations:** Clinic of Intensive Care Medicine, University Hospital Basel, Basel, Switzerland; Division of Clinical Neurophysiology, Department of Neurology, University Hospital Basel, Basel, Switzerland; University of Basel, Basel, Switzerland; Division of Infectious Diseases and Hospital Epidemiology, University Hospital Basel, Basel, Switzerland

## Abstract

**Introduction:**

Acute-phase proteins, such as procalcitonin (PCT), C-reactive protein (CRP) and albumin, may relate with course and outcome in status epilepticus (SE), as seizures bring about inflammation, changes of cytokine levels and blood–brain barrier breakdown. We aimed to determine the predictive value of serum levels of PCT at SE onset for the emergence of infections and unfavorable outcome in adult patients with SE. Furthermore, we sought to compare the predictive value of PCT, CRP and albumin for death.

**Methods:**

This observational cohort study was performed in the intensive care units of the University Hospital Basel (Switzerland), a university-affiliated tertiary care center. Adult patients with SE admitted from 2005 to 2012 were included. Serum levels of PCT, CRP and albumin were assessed at SE onset. Unfavorable outcome (i.e., death and a Glasgow Outcome Score of 1 to 3) during hospital stay and mortality after 30 days were considered the primary and infections as the secondary outcome measures.

**Results:**

In 91 SE patients, mortality was 23.1 % during hospital stay and at 30-days follow-up. Infections emerged in 30.8 % of patients. In the multivariable analysis, PCT predicted unfavorable outcome independently from possible confounders such as acute etiology, infections during SE, the Charlson Comorbidity Index, and the Status Epilepticus Severity Score (hazard ratio 1.44 per every increasing ug/L, 95 % confidence interval 1.11-1.87). Additional multivariable analysis including serum levels of PCT, CRP and albumin revealed PCT as the only biomarker independently associated with an increased hazard for unfavorable outcome. PCT levels at SE onset were not related to infections during SE.

**Conclusions:**

Serum PCT levels measured at SE onset are independently associated with unfavorable outcome but do not predict the emergence of infections during SE. Procalcitonin may increase the predictive value of clinical scoring systems allowing for rapid risk stratification early in the course of SE.

## Introduction

Status epilepticus (SE) is a life-threatening emergency encompassing a heterogeneous group of disorders with various prognoses and high mortality of up to 40 % [1]. Predicting outcome is difficult and based on clinical and electrophysiological determinants including the patient’s age, SE etiology, seizure history, level of consciousness during SE, and initial seizure type [2]. The status epilepticus severity score (STESS) relies on the strongest and most readily available outcome predictors to predict mortality early in SE, consisting of the following variables: history of seizures, age, seizure type, and impairment of consciousness [3]. External validation indicates that STESS performs well in different cohorts, but the cutoff point for survival versus death differs among cohorts [4]. Recently, the epidemiology-based mortality score in status epilepticus was presented, using a complex scoring system with a combination of etiology, age, comorbidities, and features on electroencephalography (EEG) [5]. However, EEG is not universally available or may be deemed unnecessary and the assessment of seizure history at SE onset is limited to patients who are awake, or who are in the presence of relatives. Thus, such complex scoring systems are of limited (or academic) use. An ideal prognostic tool in SE would rely on simple and ubiquitous parameters. In intensive care, a reliable prognostic tool would be very helpful, as critically ill patients identified with imminent poor prognosis would profit from early escalation of antiepileptic treatment, intensified continuous video-EEG monitoring, or neuroprotective measures, especially as patients with more benign types of SE are at risk of being over-treated.

Experimental and clinical studies have led to a growing body of evidence that seizures cause systemic inflammation, which may vice versa trigger or sustain seizures [6, 7]. A study of serum concentrations of acute-phase proteins measured early in SE revealed an independent association between albumin serum levels and treatment refractory SE as well as death, while C-reactive protein (CRP) levels were inconsistent [8]. Another promising acute-phase protein is procalcitonin (PCT), a pre-propeptide precursor of the thyroid hormone calcitonin secreted from the thyroid parafollicular cells, which increases under various inflammatory conditions, most notably with bacterial infections and sepsis [9, 10]. PCT is more accurate than CRP in diagnosing infections, especially sepsis [11, 12] and studies have identified associations between PCT and poor outcome or death in patients with alterations of the central nervous system, such as ischemic stroke [13] and postanoxic encephalopathy [14]. Despite these findings, analyses of the diagnostic and prognostic yield of PCT in patients with SE are scarce and restricted to the diagnosis of infections emerging during SE in small numbers of patients [15].

The aim of this study was to determine the predictive value of serum levels of PCT at SE onset for the emergence of infections and unfavorable outcome as determined by the Glasgow outcome scale (GOS) in adult patients with SE. Furthermore, we sought to compare the predictive value of PCT and other acute-phase proteins such as CRP and albumin for unfavorable outcome.

## Methods

### Setting and ethics

This study was performed at the University Hospital Basel, an academic medical care center in Switzerland. Subjects included are part of an ongoing prospective database of adult SE patients (≥18 years of age) treated in ICUs between 2005 and 2012 [16]. In our institution, acute-phase reactants such as PCT, CRP and albumin are routinely assessed in all ICU patients as described elsewhere [8]. All SE patients with measurements of serum levels of PCT, CRP and albumin within the first 24 hours of SE onset were included in order to compare the diagnostic and predictive power of all three acute-phase proteins. In our institution, all SE patients are treated in the ICU according to the current international guidelines [17]. All ICUs requested consultations from the same team of neurologists on the diagnosis and treatment of SE patients, and followed the same treatment algorithm according to the international and Swiss guidelines. We adhered to the strengthening the reporting of observational studies in epidemiology (STROBE) statement guidelines for reporting observational studies [18].

The study was approved by the local ethics committee (Ethikkommission Beider Basel (EKBB)) as part of the quality assurance program, and informed consent was waived because all interventions were part of standard patient care.

### Patients and data collection

All data used in this study were recorded in digital ICU and EEG databases during patients’ care. Patients with SE from hypoxic-ischemic encephalopathy after cardiac arrest were excluded. SE was confirmed clinically and/or by EEG. All EEG recordings were interpreted by two Board-certified epileptologists and consensus diagnosis was reached after review. Clinically suspected SE was confirmed by detection of epileptic seizures with EEG, lasting more than 5 minutes or episodes of more than 5 minutes with recurrent seizures and no complete neurofunctional recovery in between, according to published recommendations [17].

Level of consciousness, seizure history and worst seizure type were assessed in order to perform gradation of SE severity by the STESS as described elsewhere [3]. Seizure types were categorized into three groups with different severity scores: simple partial, complex partial, and absence seizures (not severe), followed by generalized convulsive seizures (moderate severity), and nonconvulsive SE in coma (marked severity).

Etiology of SE was categorized as suggested by the International League Against Epilepsy (ILAE) as follows: (1) acute symptomatic SE was defined as SE resulting from brain tumors, intracranial hemorrhage, acute cerebral infarct (<1 week before SE), intoxication or drug withdrawal, traumatic brain injury (not older than 1 week), encephalitis, metabolic derangements, and brain surgery <1 week before SE; (2) remote symptomatic SE included etiology such as static brain pathology, brain surgery >1 week before SE, and old cerebral infarct (>1 week before SE); (3) symptomatic SE due to progressive brain disorders was defined as SE from autoimmune and neurodegenerative brain disorders; (4) unprovoked SE of unknown etiology included patients with SE in association with idiopathic or cryptogenic epilepsy or epilepsy of unknown etiology.

The period between the time of SE onset and the time of first EEG without epileptic activity, and simultaneous absence of clinical manifestations, was defined as SE duration. As described previously [19], infections were assessed by a comprehensive review of medical charts and the database of the infection control microbiology surveillance in order to obtain all microbiological data and to cross-check microbiological findings with clinical data. A protocol for monitoring infections was established for all patients in the ICU as described previously [19]. Infections were diagnosed based on the clinical examination, radiological findings, laboratory tests, and microbiological results according to the Centers for Disease Control and Prevention criteria [20].

The outcomes assessed for this study were death and GOS at hospital discharge. A GOS of 1 to 3 (with 1 = death, 2 = persistent vegetative state, 3 = severe neurofunctional disability, 4 = moderate neurofunctional disability, and 5 = survival with mild or no disability) was classified as an unfavorable outcome.

Most surviving SE patients are scheduled for follow-up visits in our institution. We were able to capture all inpatient and outpatient medical visits within the University Hospital Basel allowing the assessment of 30-day mortality.

### Measurements of serum levels of procalcitonin (PCT), C-reactive protein (CRP) and albumin

PCT was measured with a high sensitivity time-resolved amplified cryptate emission (TRACE) technology assay (PCT Kryptor®, B.R.A.H.M.S. AG, Hennigsdorf, Germany). The assay has a detection limit of 0.02 μg/L and functional assay sensitivity of 0.06 μg/L, which is, 3-fold to 10-fold above normal mean values. CRP concentrations were determined by an enzyme immunoassay with a detection limit of 0.5 mg/L (EMIT; Merck Diagnostica, Switzerland). Albumin concentrations were measured with the pH indicator bromcresol purple (Modular Analyzer; Roche Diagnostics, Switzerland).

### Treatment characteristics

The number of antiepileptic drugs (AEDs), the use of continuously administered anesthetics to induce therapeutic coma, and use of mechanical ventilation was noted. The treatment algorithm was standardized according to the international guidelines throughout the entire study period as previously published [16]. In short, the treatment was as follows: initial treatment with first-line AEDs (intravenous benzodiazepines administered as bolus), followed by second-line AEDs (including phenytoin, levetiracetam, and valproic acid) if SE persisted, and further escalated with non-anesthetic third-line AED treatment (including lacosamide, topiramate, vigabatrin, carbamazepine, and oxcarbazepine), and continuously administered anesthetic drugs (including continuous infusions of midazolam, propofol, and barbiturates) if second-line AEDs failed.

### Outcome definition

Unfavorable outcome (i.e., death and a GOS of 1 to 3) during hospital stay and mortality after 30 days were considered the primary and infections as the secondary outcome measures.

### Statistical analysis

Patients were categorized as survivors and non-survivors. The Shapiro-Wilk test was used to distinguish between normal and abnormal distributions. The chi-square and Fisher’s exact test were applied where appropriate for comparison of proportions. Continuous variables were analyzed with the Mann–Whitney *U* test if they were not normally distributed.

Multivariable analysis was performed using the Cox proportional hazards model to calculate the hazard ratios. Variables included in this model were chosen as they were available at SE onset and either differed significantly between survivors and non-survivors in univariable comparisons or are well-established outcome predictors in SE, both representing possible confounders for the association of PCT with death and GOS 1–3. Subsequently, a multivariable Cox proportional hazards model was used to analyze the hazard ratio for death and GOS 1–3 in relation to the serum concentrations of all three acute-phase proteins (i.e., PCT, CRP and albumin). To dissociate the inflammatory effect of seizures from concomitant infection, we conducted sensitivity analysis, repeating the multivariable analysis after removing patients with SE of infectious etiology from the dataset. Interaction terms were fit to Cox proportional hazards models evaluating the association between PCT and death, and GOS 1–3 by presence of infections during SE and different types of seizures at SE onset, to evaluate effect modification. Schoenfeld residuals were determined to examine the proportional hazards assumption. Relative risks instead of hazard ratios were chosen to calculate the cumulative relative risk for having an infection during SE rather than instantaneous risks over the entire study period. Two-sided *p* values ≤0.05 were considered significant. Analyses were performed using STATA Statistical Software version 12.0 (Stata Corp., College Station, TX, USA).

## Results

Out of 303 patients with SE, 58 patients with hypoxic-ischemic encephalopathy and 154 patients without measurements of acute-phase proteins (i.e., serum levels of PCT, CRP and albumin) within the first 24 hours after SE onset were excluded. Univariable comparisons were performed to identify differences between the included and excluded SE patients without hypoxic-ischemic encephalopathy. Patients included in this study did not differ from the 154 excluded patients in the severity of SE, duration of SE, or comorbidities. Patients included in our study, however, were younger as compared to excluded patients (included patients had median age 62.1 years, IQR 48.2 − 74.0 versus median age 67.7 years, IQR 56.2 − 76.4 in excluded patients; *p* = 0.033).

Of the 91 patients included in the analysis, 21 patients died. None of the patients had liver dysfunction or failure that might have influenced serum PCT levels. Univariable comparisons of demographics, clinical characteristics at SE onset and during the course of SE in survivors and non-survivors are presented in Table 1. Non-survivors had more severe SE (according to STESS), a higher Charlson comorbidity index, longer duration of SE, and were more often mechanically ventilated.

**Table 1:** Comparisons of demographics and clinical features in survivors and non-survivors (n = 91)

	Survivors (n = 70)		Non-survivors (n = 21)		*P* value
Demographics					
Male, n (%)	32	(45.7)	11	(52.4)	0.591
Age, years, median (IQR)	60.6	(48.1 − 73.9)	65.3	(58.8 − 74.3)	0.451
Clinical features known at status epilepticus onset					
ᅟStatus epilepticus etiology grouped according to the ILAE^1^, n (%)					0.241*
ᅟAcute symptomatic seizures	37	(52.9)	14	(66.7)	
ᅟRemote symptomatic unprovoked seizures	8	(11.4)	4	(19.1)	
ᅟSymptomatic seizures due to progressive central nervous system disorders	5	(7.1)	1	(4.8)	
ᅟUnprovoked seizures of unknown etiology	20	(28.6	2	(9.5)	
ᅟInfectious etiologies of status epilepticus	5	(7.1)	1	(4.8)	
ᅟSTESS^2^ at status epilepticus onset, median (IQR)	3	(2 − 4)	4	(4 − 6)	**<0.001**
ᅟSTESS^2^ characteristics					
ᅟᅟStuporous/comatose, n (%)	38	(54.3)	16	(76.2)	
ᅟᅟWorst seizure type, n (%)					
ᅟᅟᅟSimple partial/complex/absence	36	(51.4)	5	(23.8)	
ᅟᅟᅟGeneralized convulsive	13	(18.6)	1	(4.8)	
ᅟᅟᅟNonconvulsive status epilepticus in coma	21	(30.0)	15	(71.4)	
ᅟᅟAge ≥65 years, n (%)	31	(44.3)	12	(57.1)	
ᅟᅟNo history of seizures, n (%)	43	(61.4)	16	(76.2)	
ᅟCharlson comorbidity index, median (IQR)	2	(1 − 3)	4	(3 − 6)	**<0.001**
Laboratory findings					
ᅟProcalcitonin serum level at status epilepticus onset, μg/L, median (IQR)	0.18	(0.08 − 0.41)	0.58	(0.12 − 1.27)	**0.009**
ᅟC-reactive protein serum level at status epilepticus onset, mg/L, median (IQR)	18.9	(3.7 − 47.7)	26.6	(17.1 − 54.7)	0.151
ᅟAlbumin serum level at status epilepticus onset, g/L, median (IQR)	29	(24 − 34)	27	(20 − 30)	**0.050**
Course of status epilepticus					
ᅟStatus epilepticus duration, days, median (IQR)	1	(0.5 − 2)	2	(1 − 4)	
ᅟComplications during status epilepticus					
ᅟᅟInfections, n (%)	18	(25.7)	10	(47.6)	0.065*
ᅟᅟRespiratory tract infections, n (%)	16	(22.9)	8	(38.1)	
ᅟᅟBlood stream infections, n (%)	4	(5.7)	2	(9.5)	
ᅟᅟUrinary tract infections, n (%)	2	(2.8)	3	(14.3)	
ᅟᅟHemodynamic instability requiring vasopressors, n (%)	5	(7.1)	4	(19.1)	
Treatment during status epilepticus					
ᅟAntiepileptic drugs, median (IQR)	3	(2 − 4)	4	(3 − 5)	0.423
ᅟUse of anesthetic drugs, n (%)	34	(48.6)	15	(71.4)	0.083*
ᅟMechanical ventilation, n (%)	41	(58.6)	18	(85.7)	0.035*

^1^Grouping of etiologies according to the guidelines of the International League Against Epilepsy (ILAE) [52]. ^2^STESS = status epilepticus severity score including age, level of consciousness, worst seizure type, and seizure history [3, 4]. *Fisher’s exact test; Bold *p* values are considered significant

### Acute-phase proteins and unfavorable outcome during hospitalization

**Fig. 1:**
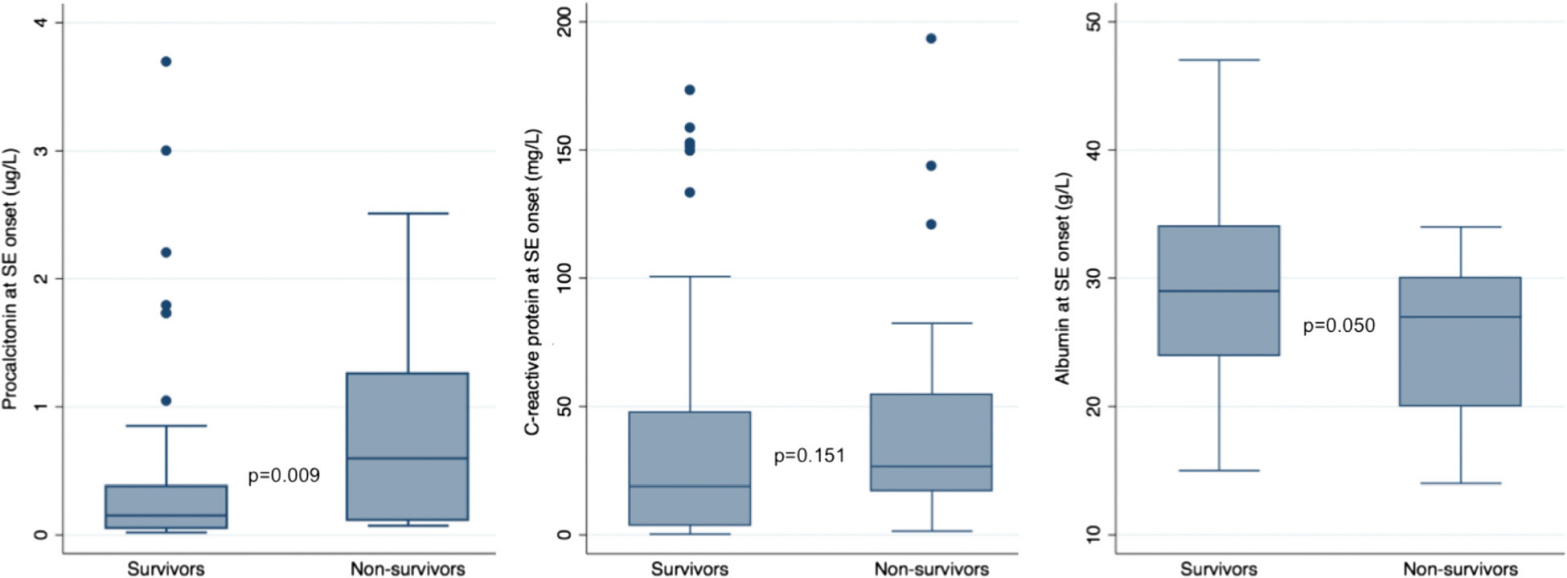
Serum levels of acute-phase proteins at onset of status epilepticus (*SE*)

Serum levels of PCT were significantly higher, but serum levels of albumin were significantly lower in non-survivors (Table 1). Serum levels of CRP were higher in non-survivors than in survivors, but the difference was not statistically significant. Figure 1 presents the serum levels of the three acute-phase proteins in survivors and non-survivors.

A multivariable Cox proportional hazards model was performed including PCT, acute SE etiology, infections during SE, the Charlson comorbidity index, and STESS. These variables were chosen as they were available at SE onset and either differed significantly between survivors and non-survivors in univariable comparisons or are well-established outcome predictors in SE, both representing possible confounders for the association of PCT with unfavorable outcome (Table 2). Thereby, PCT remained significantly associated with unfavorable outcome independently of these possible confounders. Analysis of Schoenfeld residuals detected insignificant *p* values, consistent with the proportional hazards assumption (for death: *χ*^*2*^*6.54*, *p* = 0.257; for GOS 1–3: *χ*^*2*^*2.94*, *p* = 0.709).

**Table 2:** Multivariable analyses for the predictive value of PCT for death, adjusting for potential confounders

	Total cohort			Patients without SE of infectious etiology		
	HR	95 % CI	*P* value	HR	95 % CI	*P* value
Death						
ᅟPCT at SE onset, per μg/L	1.44	1.11 − 1.87	**0.007**	1.40	1.09 − 1.82	**0.010**
ᅟAcute etiology of SE^1^	0.54	0.19 − 1.55	0.255	0.65	0.23 − 1.82	0.411
ᅟInfections during SE	3.04	1.20 − 7.71	**0.019**	3.45	1.32 − 9.00	**0.011**
ᅟCharlson comorbidity index, per unit	1.35	1.09 − 1.67	**0.005**	1.27	1.02 − 1.57	**0.030**
ᅟSTESS, per unit^2^	1.39	1.03 − 1.88	**0.035**	1.48	1.07 − 2.03	**0.017**
GOS 1 − 3						
ᅟPCT at SE onset, per μg/L	1.29	1.02 − 1.62	**0.032**	1.29	1.03 − 1.61	**0.027**
ᅟAcute etiology of SE^1^	0.59	0.34 − 1.03	0.062	0.68	0.38 − 1.22	0.196
ᅟInfections during SE	1.67	0.98 − 2.85	0.059	1.68	0.97 − 2.94	0.066
ᅟCharlson comorbidity index, per unit	1.05	0.93 − 1.18	0.466	1.01	0.89 − 1.15	0.845
ᅟSTESS, per unit^2^	1.04	0.89 − 1.22	0.628	1.06	0.90 − 1.24	0.498

^1^Acute etiology was defined as brain tumors, intracranial hemorrhage, acute cerebral infarct (<1 week before SE), intoxication or drug withdrawal, traumatic brain injury (<1 week before SE), encephalitis, metabolic derangements, and brain surgery <1 week before SE according to the guidelines of the International League Against Epilepsy (ILAE) [52]. ^2^STESS = status epilepticus severity score, including age, level of consciousness, worst seizure type, and seizure history [3, 4]. *HR* hazard ratio, *PCT* procalcitonin, *SE* status epilepticus, *GOS* Glasgow outcome score; Bold *p* values are considered significant

To compare the predictive value of the different acute-phase proteins, an additional Cox proportional hazards model was performed including PCT, CRP and albumin (Table 3). Thereby, PCT remained the only biomarker independently associated with an increased HR for death of 1.52 for every increasing microgram (95 % CI 1.17 − 1.98) and an increased HR of 1.30 for every increasing microgram (95 % CI 1.01 − 1.68). Analysis of Schoenfeld residuals detected insignificant *p* values, consistent with the proportional hazards assumption (for death: *χ*^*2*^ 0.57, *p* = 0.903; for GOS 1–3: *χ*^*2*^ 2.56, *p* = 0.464).

**Table 3:** Multivariable analyses for the predictive value of different acute-phase proteins for death

	Total cohort			Patients without SE of infectious etiology		
	HR	95 % CI	*P* value	HR	95 % CI	*P* value
Death						
ᅟPCT at SE onset, per μg/L	1.52	1.17 − 1.98	**0.002**	1.52	1.18 − 1.96	**0.001**
ᅟCRP at SE onset, per mg/L	1.01	1.00 − 1.01	0.229	1.01	1.00 − 1.01	0.268
ᅟAlbumin at SE onset, per g/L	0.99	0.93 − 1.08	0.825	1.01	0.94 − 1.08	0.790
GOS 1 − 3						
ᅟPCT at SE onset, per μg/L	1.30	1.01 − 1.68	**0.041**	1.30	1.02 − 1.67	**0.033**
ᅟCRP at SE onset, per mg/L	1.00	0.99 − 1.01	0.085	1.00	1.00 − 1.01	0.271
ᅟAlbumin at SE onset, per g/L	1.03	0.99 − 1.07	0.082	1.03	0.99 − 1.07	0.141

*HR* hazard ratio, *PCT* procalcitonin, *CRP* C-reactive protein, *SE* status epilepticus, *GOS* Glasgow outcome score; Bold *p *values are considered significant

Sensitivity analyses, repeating the multivariable analyses after excluding patients with infectious SE etiology, revealed similar results for the association between PCT and the outcome measures (Tables 2 and 3).

### Acute-phase proteins and mortality at 30-day follow up

Of the 70 patients surviving until hospital discharge, follow-up data at 30 days after SE onset were available for 61 patients (87.1 %): no additional deaths were recorded. Of the nine patients lost to follow up, four patients were discharged (to return home without additional care) or transferred to another hospital in stable conditions. In-hospital and 30-day (after SE onset) mortality was 23.1 %. Results from multivariable analysis of the predictive value of clinical variables and acute-phase protein serum levels did not differ at 30-day follow up as compared to the in-hospital outcomes presented above (data not shown).

### Effect modification by infections, etiologies and seizure types

In order to evaluate effect modification of the predictive value of serum PCT levels for death by the presence of infections during SE, different types of seizure at SE onset and different SE etiologies, interaction terms were fit to the Cox proportional hazards models. Neither the presence of infection during SE, nor specific types of seizure at SE onset, or acute SE etiologies had a significant effect on the association between elevated serum PCT levels and death (interaction terms for infection, *p* = 0.788; interaction terms for types of seizure at SE onset, *p* = 0.958; interaction terms for acute SE etiologies, *p* = 0.908).

### Acute-phase proteins and infections

A total of 28 patients (30.8 %) developed one or more infections during SE. Among those, the most common infections were respiratory tract infections (n = 24, 85.7 %), in which a microbiological diagnosis was made in 18 patients. The second most common types of infection were bacteremia (n = 6, 21.4 %) and urinary tract infection (n = 5, 17.9 %), all diagnosed with microbiological identification of a causative pathogen. One patient suffered from Herpes simplex virus encephalitis. Serum levels of PCT and CRP at SE onset did not predict emergence of infections during SE (PCT: RR_(per μg/L)_ 1.04, 95 % CI 0.80 − 1.36; CRP: RR_(per mg/L)_ 1.00, 95 % CI 0.99 − 1.01).

## Discussion

To our knowledge, this is the first study quantifying the association between serum levels of PCT measured at SE onset, and the emergence of infections and outcome in SE, in comparison with other acute-phase proteins such as CRP and albumin. While serum levels of PCT at SE onset were not significantly associated with the emergence of infections during SE, the increase of serum PCT levels early in the course of SE was significantly associated with a high hazard for death and a GOS of 1–3, independent of strong outcome predictors, such as SE severity, acute SE etiology, infections during SE, the Charlson comorbidity index, and the STESS. Further, there was no significant effect modification of the association between increased PCT levels and death or a GOS of 1–3, by infections, different seizure types at SE onset, or SE of acute etiology. In addition, PCT was the only acute-phase protein that remained highly significantly associated with death and a GOS of 1–3 among other acute-phase proteins measured at SE onset. The hazards presented in this study may seem small, however, it has to be taken into account that they are given for each change of 1 ug/L in PCT, for each increase of 1 mg/L in CRP, and for each decrease of 1 mg/L in albumin, respectively. Thus, in an individual SE patient an elevated serum PCT of 1.1ug/L has a hazard of 1.65 for death as compared to SE patients with normal serum PCT levels.

In summary, our data confirm previous findings from a study in a smaller cohort [15] that serum PCT levels at SE onset are unreliable for the diagnosis of infections, but that they are associated with prognosis in adult patients with SE, independently and more reliably than other acute-phase proteins.

Although patients included in our cohort were significantly younger as compared to patients excluded from this study, baseline characteristics in our study cohort are similar to those in prior studies of SE. In particular, median age in our cohort was similar to that in prior SE studies [21, 22], the proportion of infections during SE lies in the range of other studies of SE patients [19, 23], and mortality is close to that in population-based studies [24]. The proportions of acute etiologies were comparable to those in other SE cohorts [21, 22]. Nonconvulsive SE was slightly more prevalent than in most studies [21, 22], well-explained by the heightened awareness of nonconvulsive seizures and the increasing use of continuous EEG monitoring in our ICUs [25]. The selection of our patients with early measurements of acute-phase proteins may impede generalizability of our results calling for further prospective studies. However, none of the examined characteristics differed significantly between the selected and excluded patients (with the exception of age). Further, it seems very unlikely that additional variables were missed that would have significantly changed not only acute-phase reaction but also unfavorable outcome and the correlation between unfavorable outcome and the level of acute-phase reactants. Taking these considerations into account, further bias appears to be negligible.

The interplay among acute-phase proteins, infections and outcomes in patients with SE may be elucidated by recent findings, focusing on the interrelationships of systemic inflammatory reactions and epileptic activity [6, 7]. During acute inflammation, cytokines, such as interleukin-1 (IL-1), IL-6, and tumor necrosis factor-alpha (TNF-alpha), are released, which subsequently induce acute-phase responses [26]. There is a growing body of evidence for the value of acute-phase proteins to predict mortality in the early phase of diseases in neurocritically ill patients.

Serum albumin, a negative acute-phase protein, has been shown to decrease by about 20 % in inflammatory states [26, 27]. Low serum albumin has been identified as a predictor of death in acute ischemic stroke [28], underscoring the impact of acute inflammatory reactions in critically ill patients. The results of our univariable comparisons between survivors and non-survivors in the present study confirm our previous findings that low serum albumin is a predictor of mortality in SE [8]. However, PCT was not included in our prior study. In our current multivariable model, including albumin, CRP, and PCT serum levels, albumin did not remain an independent predictor of death and a GOS of 1–3; these results are most likely explained by the fact that in our current multivariable model PCT was added as a new predictor, which overpowered the predictive value of albumin and CRP serum levels.

Increasing levels of serum CRP have been described to indicate growing intracerebral hematomas [29] and mortality in patients with intracerebral hemorrhage [30], and to predict poor neurofunctional outcome and death in patients with acute ischemic stroke [31]. However, external validation of these finding with prospective studies are pending, and the predictive value of CRP for outcome after ischemic stroke remains controversial, as other studies did not confirm these associations [32]. The evidence for the predictive value of CRP for outcome in SE patients has been scarce so far and is mostly restricted to a few studies in children [33, 34]. The present study demonstrates that in adult patients suffering from SE, CRP does not reliably predict death, a GOS of 1–3, or infectious complications.

PCT has been reported as a biomarker for outcome in patients with ischemic strokes [13] and postanoxic encephalopathy [35]. However, the predictive value for outcome in SE patients has not been evaluated. The fact that in our study, increasing PCT serum levels were not significantly associated with the emergence of infections during SE but with mortality, indicates that the acute-phase response from PCT, which is likely to be triggered by prolonged seizures, overpowers the PCT response induced by infection. This finding is supported by previous investigations of the interplay of immunological reactions during epileptic activity [6, 7]. We cannot exclude that in some cases infections were present but not detected and that the limited sample size may have influenced our results. However, the latter seems unlikely, as the confidence intervals in our results are small. The possible promoting influence of specific underlying acute etiologies, including ischemic stroke and traumatic brain injury on increasing PCT serum levels in our study was addressed by adjusting for acute etiologies of SE in our multivariable model including these entities. As there is a large overlap of low measurements of PCT between survivors and non-survivors, our results do not indicate that a lower PCT level has a predictive value for outcome.

In recent years clinical and experimental research has uncovered several complex mechanisms as integral parts of a bidirectional relationship between SE and inflammation. Systemic and local inflammation caused by underlying etiologies or the cytotoxic effect of the accumulating excitatory neurotransmitter glutamate as the result of ongoing seizures may contribute to sustained epileptic activity [36–38]. Induction of systemic inflammatory reactions by prolonged epileptic seizures is mirrored by changes in cytokine levels, increases in circulating immune cells (e.g., neutrophils, lymphocytes, and natural killer cells), and disruption of the blood–brain barrier [38, 39], independently of emerging infections [6]. The seizure-promoting effect of inflammation and its influence on the outcomes of SE patients has been demonstrated in recent studies. Increases in cytokine levels are reflected by higher production of IL-1beta, IL-2, IL-6, and TNF-alpha by peripheral blood mononuclear cells in epileptic patients than in controls [40]. In addition, a significant increased serum level of IL-6, and total numbers of leukocytes, lymphocytes, neutrophils, and natural killer cells were measured during the first minutes of the postictal phases after seizures in patients with temporal lobe epilepsy [39]. This systemic and focal inflammatory reaction may alter the blood–brain barrier and increase permeability for ions and proteins, like albumin, facilitating transmigration of inflammatory cells that contribute to sustained epileptic activity [41]. Modulation of neuronal activity and viability by cytokines that promote the release of neuroactive molecules from glia or the endothelium (e.g., glutamate, nitric oxide, neurotrophins) [42, 43], or by activating neuronal receptors in the central nervous system [44, 45] has been described. In addition, neurons and glial cells may express functional cytokine receptors in vitro [46, 47] and in vivo [48], underscoring that both the immune system and the central nervous system have a bidirectional influence on each other. Although cytokines have several physiological functions, including the induction or maintenance of neurogenesis, neurite outgrowth and synaptic pruning during development of the central nervous system [49], their exaggerated release and persistence in the brain can lead to neuronal dysfunctions that may result in seizures or epilepsy [50, 51].

Taking these and our results into account, a bidirectional influence of SE and systemic inflammation as reflected by alterations of acute-phase proteins and, consecutively, the correlation between acute-phase proteins and SE outcome become more than plausible. Although in our analyses the Charlson comorbidity index and STESS were additional independent outcome predictors, their assessment at SE onset is challenging, as they depend on patients’ history, a variable that cannot be determined if patients have altered mental status and relatives are absent. In contrast, serum PCT is a promising and readily available biomarker that may increase outcome prediction in patients with SE once integrated into future prediction models. However, as our findings are the result of observations in a single-center cohort, no clinical extrapolation of our results can be made at this time, and our data need to be carefully validated in prospective external and independent cohort studies.

### Limitations

Although most baseline characteristics in our study population are comparable to those in other studies of SE and the results seem plausible in light of the evidence mentioned above, there are limitations, mainly related to the observational single-center design. In addition, the results regarding the 30-day mortality have to be interpreted with caution, as we lost 12.9 % of patients to 30-day follow up. However, as four of the nine patients lost to follow up were discharged in stable conditions, it seems unlikely that this would have significantly influenced our results for 30-day mortality. Subgroup analyses of the possible effect modification of the association between increased serum PCT levels and death or a GOS of 1–3 by infections, different seizure types at SE onset, and acute etiology, may be hampered by the limited sample size. However, we were not able to identify any trends towards a likely interaction between any of the examined variables and the association between PCT and death. Sensitivity and specificity calculation for specific cutoff points was not performed because the sample size in our study was too small for the assessment of stable point estimates of the variable.

## Conclusion

Serum PCT level measured at SE onset is independently associated with death and a GOS of 1–3, but not with the emergence of infections. Further studies are needed to strengthen our results and to assess the potential of PCT as a reliable parameter for prediction models, together with clinical findings known to be associated with severe course and poor outcome of SE.

## Key messages

- Increased serum level of procalcitonin at onset of status epilepticus is associated with unfavorable outcome independent of possible confounders
- Among different acute-phase proteins, serum levels of procalcitonin at onset of status epilepticus are stronger outcome predictors than C-reactive protein and albumin
- Serum levels of procalcitonin at onset of status epilepticus did not predict the emergence of infection in patients with status epilepticus

## Abbreviations

AED: antiepileptic drug
CI: confidence interval
CRP: C-reactive proteins
EEG: electroencephalography
GOS: Glasgow outcome score
HR: hazard ratio
ICU: intensive care unit
IL: interleukin
ILAE: International League Against Epilepsy
PCT: procalcitonin
SE: status epilepticus
STESS: status epilepticus severity score
